# “Heidelberg Standard Examination” – Final year students' experiences with a handbook and instructional videos to improve medical competence in conducting physical examinations

**DOI:** 10.3205/zma001184

**Published:** 2018-08-15

**Authors:** Julia Knauber, Anna-Katharina König, Tobias Herion, Julia Tabatabai, Martina Kadmon, Christoph Nikendei

**Affiliations:** 1 University Hospital Heidelberg, Department of General Internal Medicine and Psychosomatics, Heidelberg, Germany; 2 University Hospital Heidelberg, Department of surgery, Heidelberg, Germany; 3 University Hospital Heidelberg, Center for Child and Adolescent Medicine, Pediatrics Clinic I, Heidelberg, Germany; 4 University Hospital Heidelberg, Centre for Infectiology, Virology, Heidelberg, Germany; 5 University Hospital Heidelberg, German Centre for Infection Research, Heidelberg, Germany; 6 University of Augsburg, Department of Medicine, Augsburg, Germany

**Keywords:** medical education, physical examination, multimodal learning

## Abstract

**Background: **The physical examination (PE) of patients is a core competence in almost all medical disciplines. The teaching materials “Heidelberger Standard Examination”, consisting of a handbook and accompanying videos, were developed with the objective of providing medical students with an innovative faculty-wide teaching and examination standard to sustainably advance students’ PE competences during medical training.

**Methods: **In a “mixed-method approach” comprising both quantitative and qualitative measures, our study examined Heidelberg University Hospital final year (FY) medical students’ use and evaluation of the individual teaching material components. Therefore, 92 FY students completed quantitative evaluation measures and ten FY students took part in individual 30-minute semi-structured interviews.

**Results:** Of the sample of n=77 students, who had completed the clinical part of their studies at Heidelberg University Hospital, 97.4% (n=75) had used the handbook and 35.0% (n=27) the accompanying videos. The teaching materials were evaluated via the common German six-point school grading system with an average mark of 1.35±0.5 for the handbook and a mark of 2.15±1.0 for the accompanying videos. Further, our results show that FY students especially valued the “Heidelberg Standard Examination” handbook as a guide and general reference work and felt the materials improved their self-perceived PE competence. Although FY students saw the accompanying video material as helpful, it was less frequently used, indicating further development potential. Overall, results reveal that FY students perceive the “Heidelberg Standard Examination” teaching program to contribute to the improvement of the quality of their PE training.

## Zusammenfassung

Die körperliche Untersuchung (KU) von Patientinnen und Patienten ist in fast allen medizinischen Fachbereichen eine wichtige Kernkompetenz. Mit den Lehrmaterialien „Heidelberger Standarduntersuchung“, bestehend aus einem Kitteltaschenbuch und Begleitvideos, wurde ein neuer Lehr-und Prüfungsstandard entwickelt, der Medizinstudierenden die Möglichkeit gibt, bereits während des Studiums eine hohe Kompetenz in der Durchführung der KU zu erlangen. In einer sowohl quantitativ als auch qualitativ angelegten Befragung nach dem „mixed-method approach“ mit Studierenden im Praktischen Jahr (PJ) am Universitätsklinikum Heidelberg wurden Nutzungsverhalten und Bewertungen einzelner Komponenten der Lehrmaterialien untersucht. Der quantitative Teil der Untersuchung erfolgte dabei anhand eines Fragebogens, der von 92 PJ-Studierenden ausgefüllt wurde. Der qualitative Teil wurde in Form von halbstandardisierten Interviews mit zehn PJ-Studierenden durchgeführt. Von den n=77 Studierenden, die den klinischen Studienabschnitt am Standort absolviert hatten, hatten 97,4% (n=75) das Untersuchungsbuch und 35,0% (n=27) die Begleitvideos genutzt. Dabei wurde das Untersuchungsbuch mit einer Durchschnittsnote von 1,35±0,5 und die Begleitvideos mit einer Durchschnittsnote von 2,15±1,0 entsprechend dem Schulnotensystem bewertet. Unsere weiteren Ergebnisse zeigen, dass insbesondere das Kitteltaschenbuch als Leitfaden und Referenzwerk von den Studierenden sehr gut angenommen wurde und zu einer starken Verbesserung der selbstwahrgenommenen Kompetenz bei der KU beitragen konnte. Das Videomaterial wurde ebenfalls als sehr hilfreich bewertet, aber bisher noch weniger genutzt, sodass hier noch Entwicklungspotential zu sehen ist. Insgesamt zeigen unsere Ergebnisse, dass das Projekt „Heidelberger Standarduntersuchung“ dazu beigetragen hat, die Qualität der studentischen Ausbildung in Bezug auf die KU aus Sicht der Studierenden nachhaltig zu verbessern.

## Introduction

In almost all medical disciplines, the competent physical examination (PE) of patients constitutes an essential component of medical practice [1]. Combined with medical history taking, the PE of patients enables physicians to make fast and effective diagnosis and therefore forms the basis for successful treatment planning [2], [3]. Due to the PE’s central role, it is essential that every graduate student is proficient in this core medical skill. Nevertheless, despite the known importance of PE skills, research on medical students shows that many have serious deficits in performing the PE and assessing resulting findings [1], [4], [5]. In order to promote an urgently needed improvement in medical students’ performance of PE techniques, a standardized procedure for teaching is required, which should be applicable in all study phases and different subject areas [6], [7]. To ensure the acceptance of such a practical clinical offer, it seems important to implement curricular as well as optional training sessions with these tools. Further, the contents of the teaching materials should be based on and correspond with audit objectives [8]. The project “Heidelberg Clinical Standards” [https://www.heidelbergerklinischestandards.de/] was launched with the aim of establishing an interdisciplinary PE standard in coordination with all clinical disciplines at the Heidelberg University Hospital [7]. With this project, we endeavored to enable all students of the Medical Faculty of Heidelberg to learn standardized examination methods in a more targeted manner by having access to a wide range of free of charge multimedia materials. We developed a practice-oriented, pocket-sized handbook [9] and corresponding instructional videos. Our aim was to make the individual examination techniques more vivid in the sense of e-learning and, thus, to improve learning processes [7]. By integrating the developed PE standards into the curriculum, we hope to sustainably improve the quality of student education and competence acquisition which will enhance patient care altogether [7]. So far, there is no information available on whether or not a standardized program for PE techniques is perceived by final year (FY) medical students to enhance their professional competences. 

Therefore, using quantitative and qualitative measures, the present study aimed to investigate Heidelberg University Hospital FY students’ assessment of

1. their context of use,
2. perceived benefit of the handbook and the accompanying videos.
3. Further, we aimed to record the impact of these teaching materials on exam preparation and the learning of practical clinical skills, as well as
4. to review the integration of the teaching materials into the curriculum.

## Methods

### Study design

In Germany, after having completed two pre-clinical and three clinical years of training, students enter their final year (FY; German: “Praktisches Jahr”, English: practical year). This consists of three four-month internships, of which one placement must be in surgery and one in internal medicine. The medical field of the third placement is chosen individually by each student. To complete their studies, medical students must pass three medical state examinations: the first one is written and practical and takes place after the pre-clinical studies. Then, before entering the final year, medical students take the second written state examination, and after completing the three internships in their final year there is a third, practical exam. We analyzed Heidelberg University Hospital FY students’ usage behavior and evaluations of the individual components of the “Heidelberg Standard Examination” handbook and the accompanying video material [https://www.heidelbergerklinischestandards.de/] in a mixed-method approach, using quantitative as well as qualitative measures [10]. The quantitative part of the study was based on a questionnaire with 43 items (see section “Measures”). Additionally, we conducted semi-structured interviews to gain a more differentiated impression of the students’ usage behavior and to evaluate the relevance of the teaching materials for the clinical stage of their medical education and FY learning.

#### Quantitative measure study sample

The quantitative part of the study was carried out in the period from November 2016 to May 2017 at Heidelberg University Hospital. The participants were recruited from different closed cohorts of students: two cohorts were at the beginning of their FY, and one cohort was in the third part of their FY, currently undergoing practical training in the field “Internal Medicine”. Students who were at the beginning of their FY rotation were recruited during a FY introductory event [11]. Students training in "Internal Medicine" were recruited during a supervision event. All participants were informed on the background of our study before taking part. Their participation was voluntary. The completion of the questionnaire took about 15 minutes.

#### Measures

The quantitative evaluation of the handbook „Heidelberg Standard Examination” and its accompanying videos was based on a questionnaire designed by our own work group. The questionnaire was critically reviewed by experts throughout the development process and underwent a pilot testing. For this purpose, a sub-sample of 10 FY students was asked to complete the questionnaire and give feedback afterwards. The final questionnaire comprised 43 items. To begin with, there were two normative questions on the current stage of their studies and their previous place of study. Then,

1. the questionnaire investigated the context of use of the teaching materials. In a first set of questions with two items, students were asked about
2.
  - previous experiences with using the materials, possible answers being “I do not know”, “I know”, “I have already used”.
  - In a second set of questions, the frequency of using the handbook and video material in four different contexts (preparation for practical work, practical work, preparation for practical and written exam) was assessed via a five-point Likert-scale (“very often” to “never”).
3. Eight items investigated the perceived benefit of the handbook and the accompanying videos via a seven-point Likert-scale (“fully agree” to “strongly disagree”). In addition, participants could specify if they had not yet had any experience at all with aspects of the materials in question.
4. Another set of questions evaluated what impact the teaching materials had on exam preparation and learning of practical clinical skills via a seven-point Likert scale (“fully agree” to “strongly disagree”). In each case, three items concerning the practical benefits (“greater autonomy”, “higher sense of security”, “fewer errors in the physical examination”) and two items each for the benefit regarding exam preparation (written state examination and practical exams during their studies) were assessed.
5. The integration of the teaching materials into the curriculum was evaluated with one item asking after the frequency of lecturers’ references to the handbook and videos during class, and one item addressing the actual use of videos in class. Both items were assessed via a five-point Likert-scale (“very common” to “never”). Finally, there was a general assessment of both the handbook and the accompanying videos via the common German six-point school grading system ranging from “1, very good" to “6 , insufficient”.

#### Quantitative analysis 

We processed the data by means of descriptive analyses with the Statistical Package for the Social Sciences (Version 21.0, SPSS Inc., Chicago, IL, USA). To obtain a comprehensive picture of the students' response behavior, we analyzed the frequency distributions. Out of the general assessment of the handbook and video materials according to the German common six-point grading system we calculated the averages. 

#### Qualitative study sample

The recruitment for the qualitative assessment was carried out simultaneously to the quantitative survey of FY students who were in the last third of their practical training in the field “Internal Medicine” at Heidelberg University Hospital. The students of this cohort were invited to voluntarily participate in the qualitative assessment of our study. Thus, the FY students who were interviewed represent a subgroup of the FY students who participated in the quantitative survey. 

#### Development of the main interview questions and conducting the semi-structured interviews

Our qualitative assessment of the FY students‘ context of use and evaluation of the teaching materials consisted of semi-structured interviews. The key questions for the interviews were based on the quality criteria of the COREQ checklist [12] and on discussions in the expert team. Considering the methodological guidelines according to Helfferich, the interviews were designed as semi-structured, including open key questions (see Table 1 (Tab. 1)), further questions and more detailed enquiries [13].

The participants were informed about the background of the survey. Their participation was voluntary. The manual-based interviews were carried out under supervision as one-on-one interviews by an interviewer experienced and trained in qualitative research. The implementation took about 30 minutes. The interviews were recorded by voice recorder and were then verbatim transcribed.

#### Qualitative evaluation of the semi-structured interviews

The qualitative content analysis of the transcribed interviews was performed using the software MaxQDA (version 12, VERBI GmbH, Berlin). Here, we followed the guidelines of the qualitative inductive content analysis [14], which means that thematic categories were not defined in advance but were developed from the content. With the aim of identifying recurring topics, all interviews were initially subjected to an open coding. Here, we extracted representative elementary units of meaning by defining single or several content-related sentences as so-called “codes” [15]. In a following step, these codes were summarized according to relevant topics for each individual participant. The themes that recurred with several participants were compared and adapted in an iterative process, until the relevant topics could be defined for all participants. Two independent investigators assigned the codes to the respective topics, discussed cases of disagreement and changed categorizations if necessary. Finally, topics that had similar contents were grouped into main and sub categories. 

## Results

### Quantitative study sample

The questionnaire was distributed to a total of 100 FY students. We received the completed questionnaire from 92 persons, which equates a response rate of 92%. 14 students were in the last third of their FY, whereas 78 participants had only just started their FY. The vast majority of respondents (83.7%) had completed the clinical part of their studies at the University of Heidelberg. The remaining 16.3% had just come to Heidelberg University Hospital for their FY. Due to our previous findings that those students, who had come to Heidelberg University Hospital from other universities, had only little experience with using the teaching materials, the following analysis only includes the information provided by the FY students who had already completed their clinical studies in Heidelberg.

#### Quantative measure results 

##### Context of use (10 items)

Nearly all of the FY students (97.4%), who had completed their clinical studies in Heidelberg, stated that they had already used the handbook before. The vast majority (85%) had regularly used the handbook to prepare for practical exams during the clinical part of their studies, specifically the OSCEs (Objective Structured Clinical Examinations) [16]. Just under half of the FY students also said they used the book often to very frequently at home to prepare for their practical work in the hospital. A third of the students used the handbook during their working day in the hospital. Less than 20% had used the handbook to prepare for the written state examination (see Table 2 (Tab. 2)). 

Just over a third (35.1%) of the FY students stated that they had already worked with the accompanying video materials. Of these students more than half had watched the videos to study for the OSCEs. Less than 15% of the students had used the videos in preparation for the practical work in hospital and less than 25% of them had used the videos in preparation for the written state examination. 

##### Useful aspects of the handbook and the accompanying videos (8 items each)

**Figure 1:**
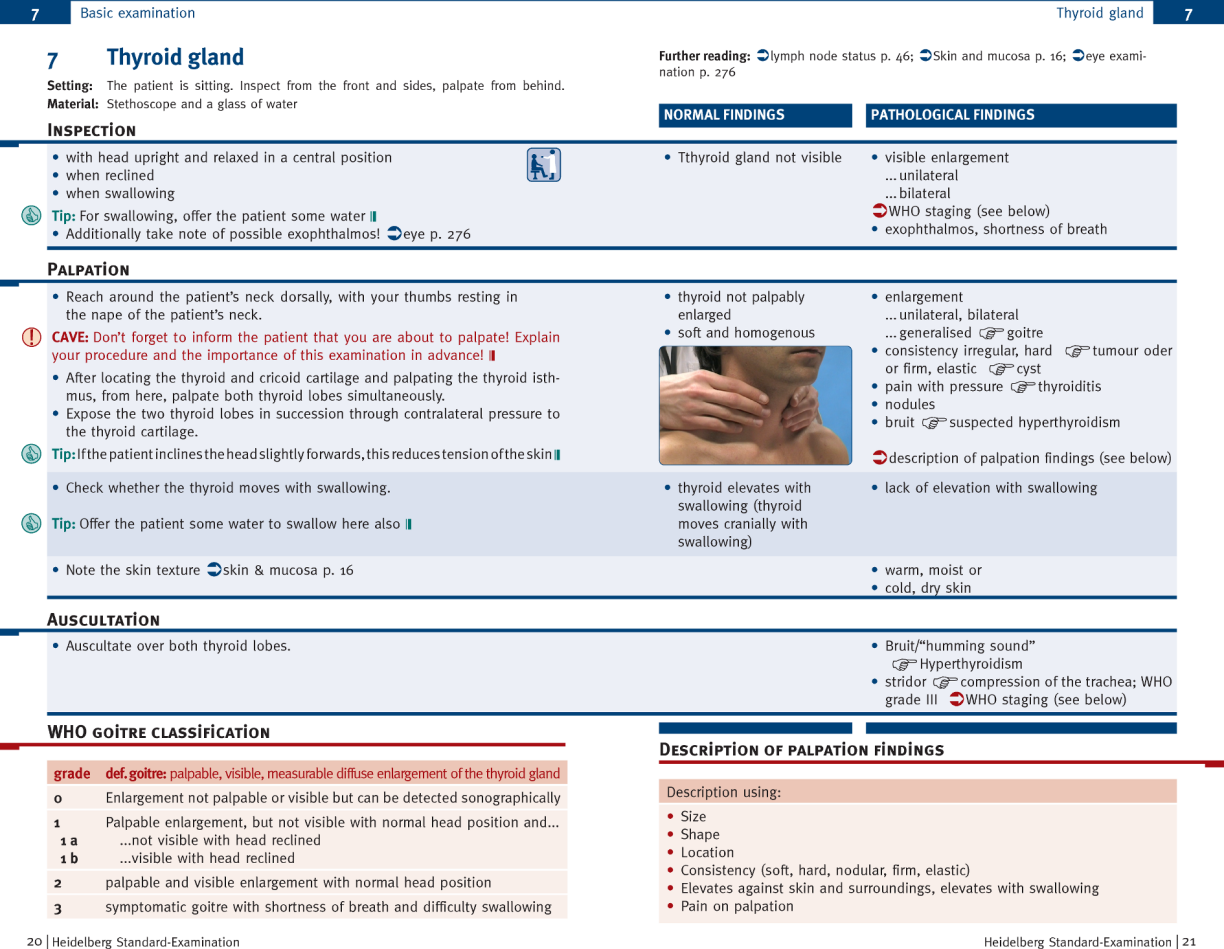
Example chapter of a basic examination from the handbook.

In answer to the question which features of the handbook they considered helpful (see Table 3 (Tab. 3)), respondents cited the step-by-step description, the schematic juxtaposition of normal and pathological findings, and the indications of possible sources of errors (CAUTIONs) and tips (for a sample chapter see Figure 1 (Fig. 1)).

As regards the accompanying videos, the speaker’s explanations, the way that the videos complemented the contents of the handbook and the integration of images were rated as particularly helpful (see Table 4 (Tab. 4)). 

##### How did using the manual and the accompanying video material influence the learning of practical clinical skills (3 items each) and the exam preparation (2 items each)?

A vast majority of FY students declared that using the handbook had led to a greater sense of security conducting the PE. One third of the respondents fully agreed with this statement, almost 39% largely agreed and 25.3% moderately agreed. Further, the students stated that because of studying with the handbook, they had felt more confident at an earlier stage to conduct the PE on their own. 28% fully agreed with this statement, 41.3% largely agreed and 26.7% moderately agreed. Moreover, the students found that they had made fewer mistakes in the PE due to using the handbook. 17.3% of them fully agreed to this, 30.7% largely agreed and 40% moderately agreed. Respondents saw the handbook as an important asset for successful preparation of the OSCEs. 60% fully agreed with this statement and another 20% largely agreed. Just under 11% of the respondents thought that the handbook had largely prepared them better for the written state examination, and around 36% found that it had been of moderate importance.

Overall, the accompanying videos were assessed in a similar manner. The students’ degree of consent concerning the statements “perceived self-confidence”, “independent implementation” and “error reduction” was mainly in the middle range. More than half of the students who had experience with the videos, thought they were mostly very helpful for the preparation of the OSCEs. However, for the preparation of the written state examination the videos played hardly any role according to the FY students (see Table 5 (Tab. 5))

##### Integrating the teaching materials into the curriculum (3 Items)

Regarding the integration of the handbook and the accompanying video materials into the university curriculum, the FY students stated that their lecturers had often referred to the handbook. However, nearly a third of the respondents declared that they had never received any information regarding the accompanying video material. Further, the majority of FY students said that they had never or only very rarely been shown the videos during classes (see Table 6 (Tab. 6)).

##### Overall evaluation of the handbook and the accompanying videos

Overall, the handbook was rated "very good" with a mean of 1.35 (0.5 standard deviation, range 1-3) according to the German school grading system. The videos were rated “good” with a mean of 2.15 (1.0 standard deviation, range 1-5).

#### Semi-structured interview sample 

The semi-structured interviews were attended by ten FY students, all of whom had already completed the clinical part of their studies at the University of Heidelberg. At the time of assessment, all the participants were in the last third of their final year. 

#### Qualitative study results 

Hereinafter, we will present the results of the content analysis of the transcribed interviews. Out of the interviews with 10 FY students, we were able to extract 203 individual statements and sort them into six main categories. Attachment 1 gives an overview of the topical areas, the associated subcategories, description of the statements and example citations. 

#### Context of use

##### Handbook use (58)

The students mainly described three different areas in which they had used the handbook during their medical studies: for exam preparation, bedside teaching and the practical work during the FY. A few students also stated that the handbook had been useful for their first practical experiences during clinical traineeships at an earlier phase in their studies.

Regarding exam preparations in the clinical part of their studies, FY students stated to have used the handbook extensively as a reference work to study for practical exams. Many of them described that they had found the handbook to be especially helpful for the OSCEs, because it provided a precise guide for the PE. Therefore, the handbook also was beneficial for the preparation for exam simulations during the FY [1] as well as for the practical second state examination. Some of the students had previously used the handbook as guidance for learning examination techniques and diagnostics in the context of bedside teaching or for revising with their fellow students. 

Within the framework of the students’ practical work during their current phase of studies, the handbook was also perceived as an important asset for preparation and follow-up of their tasks on ward. In this context, the students stated that they had found the basic examinations as well as the neurological and orthopaedic examinations in the handbook to be particularly useful for daily routines in the hospital. However, most of the respondents only noticed this significance for their practical work at the end of their studies. Very few students reported that they had already used the handbook for their practical work during the clinical part of their studies before their FY (for example during clinical traineeships).

##### Systematic classification (39)

Many students declared that they regarded the handbook as an explicit guideline for the planning of different investigations. In the context of their practical work, the respondents mentioned that with help of the handbook they could develop concepts catering to the every-day needs and routines in the hospital. Others emphasized the character of the book as a versatile reference work, especially during the FY. The students were convinced that they would continue using the handbook when starting work as assistant doctors. For many students the handbook served as a benchmark, which allowed them to have a standard for practical exams and when conferring with their lecturers. Additionally, the handbook was seen as a kind of “gold standard” for practical work. Students described that they often received diverse information from assistant doctors on how to carry out an examination correctly. With the handbook, they had an indication to what was really correct. 

Due to the abundance of information, the handbook was perceived as too “fat” and “bulky” which is why students did not explicitly see it as a book to be carried around in their pocket. However, these characteristics were not noted as negative; it was regarded as sufficient to have the handbook in the ward office and use it to prepare for upcoming examinations. Most of the students did not want to use the handbook directly at the patients’ bedside

#### Helpful aspects of the handbook

##### Positive characteristics of the handbook (44)

The majority of the FY students found the handbook’s compact and clear structure particularly helpful. They stated that it contained all essential information in a concise form and it was easy to navigate the individual pages. Despite the compact format, there was a large variety of content, so that it rarely was the case that the students were not able to find a specific examination technique. Before they had started their FY, some information had seemed rather detailed to the students; however, after having engaged more with diagnostic questioning during their practical work in the hospital, they were able to appreciate the complete account of the various specialist areas. 

For many students the graphic juxtaposition of normal and possible pathological findings was particularly convenient. Further, they found the extensive illustration useful to gain a better understanding for different examination procedures. Together with the juxtaposition of possible findings, the photographs and diagrams helped in the diagnostic assessments of clinical findings. Due to these two aspects, the students found the handbook to differ greatly from other books on the PE. The students repeatedly stressed that they had perceived the handbook as a valuable asset and that many students from other universities would also like to use such a book.

##### Criticism of the handbook (23)

While the handbook’s extensive content was praised by the students on the one hand, it was also mentioned as the most frequent point of criticism. Students found the handbook difficult to use during clinical work because too many details and different examination techniques were described. It was not feasible to perform the individual examinations in the same detailed way described in the handbook in every-day routines in the hospital. Also, it is often hard to see, which tests are more important than others. Therefore, the handbook is sometimes difficult to navigate, especially regarding the chapters on orthopaedics, neurology and paediatrics. Overall, the abundance of information leads to confusion, so that the students wished for indications in the handbook as to which aspects are essential and which are of subordinate importance, especially for members of another medical specialization. 

##### Integration of the teaching materials into the curriculum (14)

Most of the students did not remember that the handbook had been actively integrated into their classes or that it had been specifically referred to by the lecturers. They had rather used the handbook through personal initiative. However, a few of the respondents remembered that lecturers given them the hint to use the handbook in preparation of the OSCEs. Some students perceived the handbook as a kind of measure of what was expected in the OSCEs, so that it had served as an implicit basis for teaching.

##### Experiences with the accompanying videos (25)

The majority of the students declared not to have any experiences with the video material. Most of them did not feel the need to use the accompanying videos to the handbook, because they liked to watch other videos retrieved via “Google” or other websites. Even though most of them knew that the videos existed, they found it too great an effort to gain the access information. Some of the students stated that they had worked with the handbook’s first edition and had never collected the access codes that were provided later. The small number of FY students that had used the video material in the past, however, found it a useful supplement to the handbook. Watching the videos could be helpful for understanding more complex contents, such as orthopaedic examination techniques. 

## Discussion

With our project „Heidelberg Standard Examination“, we created a practical pocket-sized handbook which allows medical students to gain a high competence in the PE during their studies. By establishing a new standard for teaching and examining, we aimed to counter the development that a majority of medical students show grave deficits in performing the PE and analysing the clinical findings [1], [4], [5]. 

Our results show, that a large majority of the FY students at Heidelberg University Hospital regularly used the handbook “Heidelberg Standard Examination”. The handbook was mainly applied to learn the PE in class and to prepare for practical exams. Students were made aware of the handbook’s benefits in class. Therefore, the handbook evolved into an implicit standard reference for practical exams. Further, the handbook was regarded as an important asset to prepare the work placements in hospital during the FY and was perceived as a guideline for developing individual concepts of PE skills tailored to everyday routines. The students rated the handbook’s compact and clear structure, the graphic juxtaposition of normal and possible pathological findings, the extensive illustrations and the frequent indications of possible sources of error (CAUTIONs) as especially helpful.

Our findings are particularly important when taking into consideration that studies show that students often receive little supervision during their clinical placements concerning which aspects they must pay particular attention to during the PE [17], [18], [19]. Perhaps this deficit in practical guidance can be reduced with the implementation of the handbook. This aspect is supported by the fact that by using the handbook, students perceived themselves to be better prepared for practical exams and felt more comfortable performing the PE at an earlier stage during training. Also, they felt more secure in performing the PE and reporting to make fewer mistakes. Hence, the implementation of a central examination standard is highly conducive in furthering the FY students’ feeling of competence, even though this has meant finding compromises on what is to be seen as “standard” for the experts involved. It has been shown that teaching the PE in a skills lab and at the bedside leads to an objective enhancement of the students’ competences [20]. However, there still is a lack of controlled studies investigating the use of handbooks and teaching videos.

While the study book was very well received by the students according to the above evaluations, different usage behaviour was observed with regard to the accompanying videos. Only a little more than a third of FY students stated to have watched the handbook’s accompanying videos. Most of this group used the videos to prepare for practical exams and just a small percentage used the videos to prepare for practical tasks during their FY placements in the hospital. The students especially liked the videos’ contents complementing the handbook, the explanations and the integration of illustrations and diagrams. The students felt that the videos had furthered their competence in performing the PE. Thus, they had felt better prepared for practical exams. However, studies show how subjective perception of competence does not necessarily match objective competence [21]; further studies are needed to objectify the students’ assessments. 

Overall, the rating of the video material was less positive than the rating of the handbook. This result is quite surprising, given the fact that today’s generation of students would be “digitalized” to a large extent. The current findings also contradict other research results that have shown how the integration of teaching videos into student education leads to an increased competence in PE skills [22], [23], [24]. Possibly, our results relate to the fact that the video material was only seen as complementing the handbook and that not all students had access to the videos from the beginning due to access issues. Contrary to “blended learning” [25], which aims to combine video material shown in class directly with bedside-teaching, the integration of our accompanying video material still seems to be insufficient. A lot of students were not aware of the benefits of the videos, which is why the video material was perhaps not perceived as that helpful. Therefore, it will be important to raise the awareness for the videos in people who are in direct contact with students and can systematically integrate the videos into their classes. This could support the students’ use of this medium and further multimodal learning.

Lastly, accessing the video material on the website via a password was perceived as awkward in everyday routines. Many FY students stated that they would watch videos on Youtube™ or other websites. This corresponds to our findings that medical students and young doctors need quick and easy access to online sources [26], [27]. Therefore, it will be essential to facilitate access to the video material and, thus, achieve a wider usage of the “Heidelberg Standard Examination” video material. This could also prevent students from watching qualitatively unchecked videos which may result in learning the wrong examination techniques [28].

## Limitations

Our research project has some limitations that will be addressed in the following paragraph. One aspect is the limited number of participants. Further, there are no objective measures to determine the learning success gained through use of the handbook “Heidelberg Standard Examination” and the accompanying videos. Also, the students’ responses from the semi-structured interviews only show individual viewpoints of a small group of FY students. These students were all in their last third of the FY, studying at the University of Heidelberg. The results cannot be generalized because they do not cover all medical fields of elective placements. Especially the fact that we only questioned FY students limits originality. In addition, we did not include a survey of lecturers to investigate the actual integration of the teaching materials into classes. It would need further research projects to assess the learning success through use of the handbook and the accompanying videos in a longitudinal and standardised study design.

## Conclusion

Overall, the results of our study show that the teaching materials "Heidelberg Standard Examination" with a handbook and accompanying videos were very well received by the medical students at the University of Heidelberg. The students assessed the handbook in particular to be a standard work for learning PE skills. In their opinion using the handbook improved their practical clinical skills. Thus, our study indicates that the project "Heidelberg Clinical Standards" makes an important contribution to improve of one of the core competences during medical studies.

## Competing interests

The authors declare that they have no competing interests. 

## Supplementary Material

Analysis of the interviews conducted with FY students in the 2nd and 3rd tertial of FY medical education (n=10)

## Figures and Tables

**Table 1:**
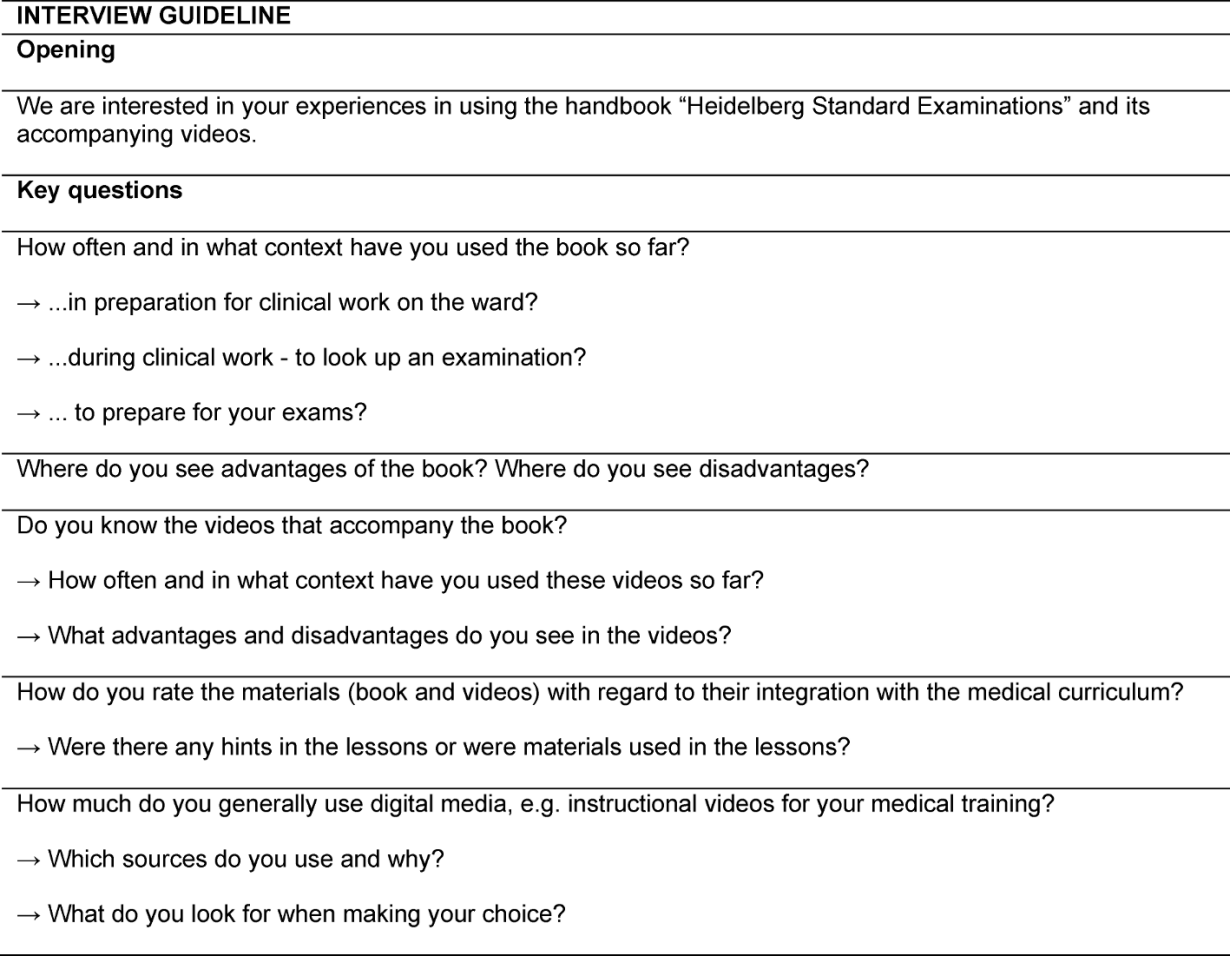
Interview guideline of the semi-standardized interviews

**Table 2:**
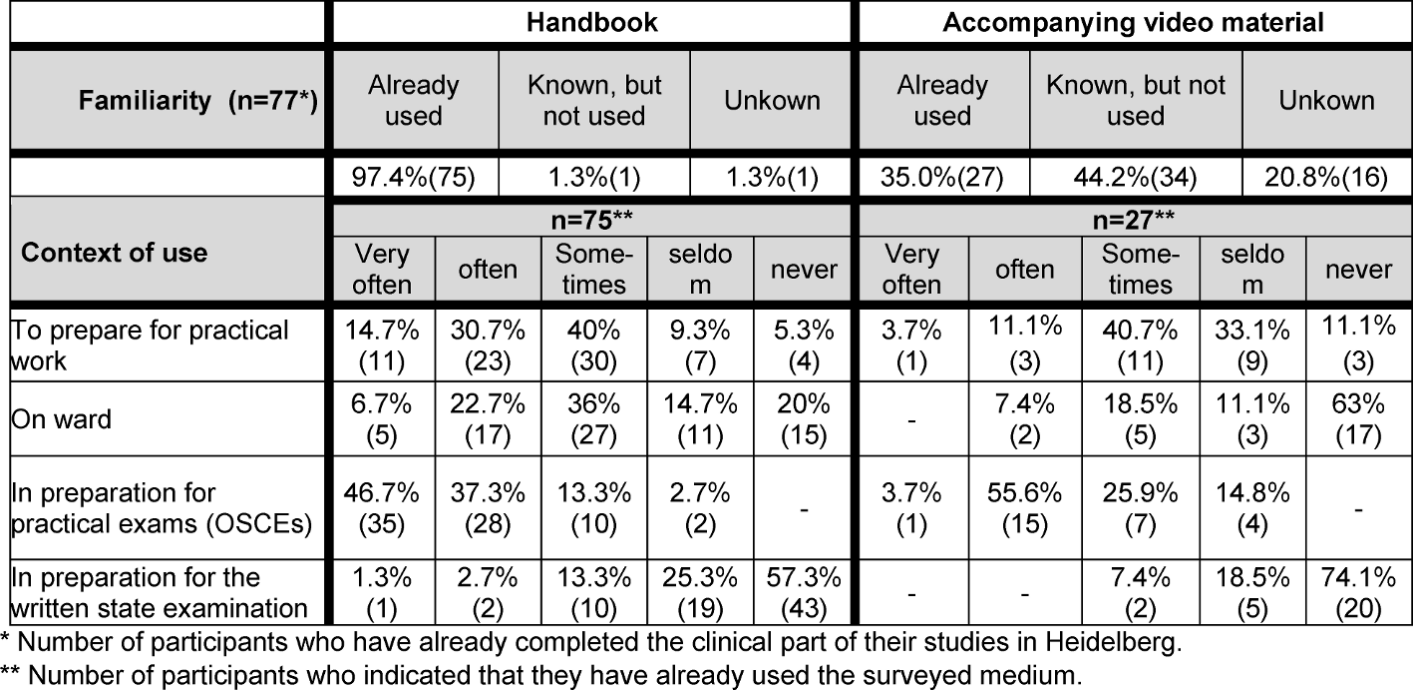
Use of the handbook and its accompanying videos: familiarity (n=77) and context of use of handbook (n=75) and the accompanying videos (n=27)

**Table 3:**
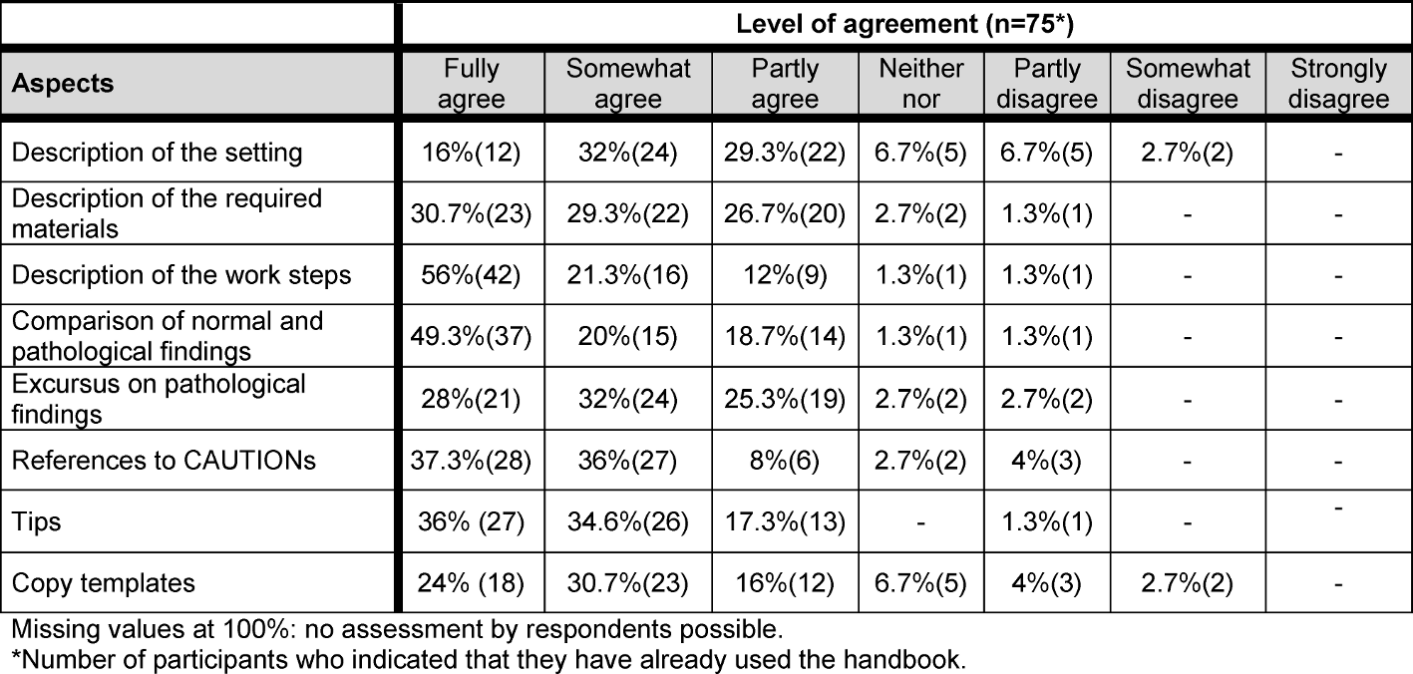
Helpful aspects of the textbook as assessed by FY students (n=75).

**Table 4:**
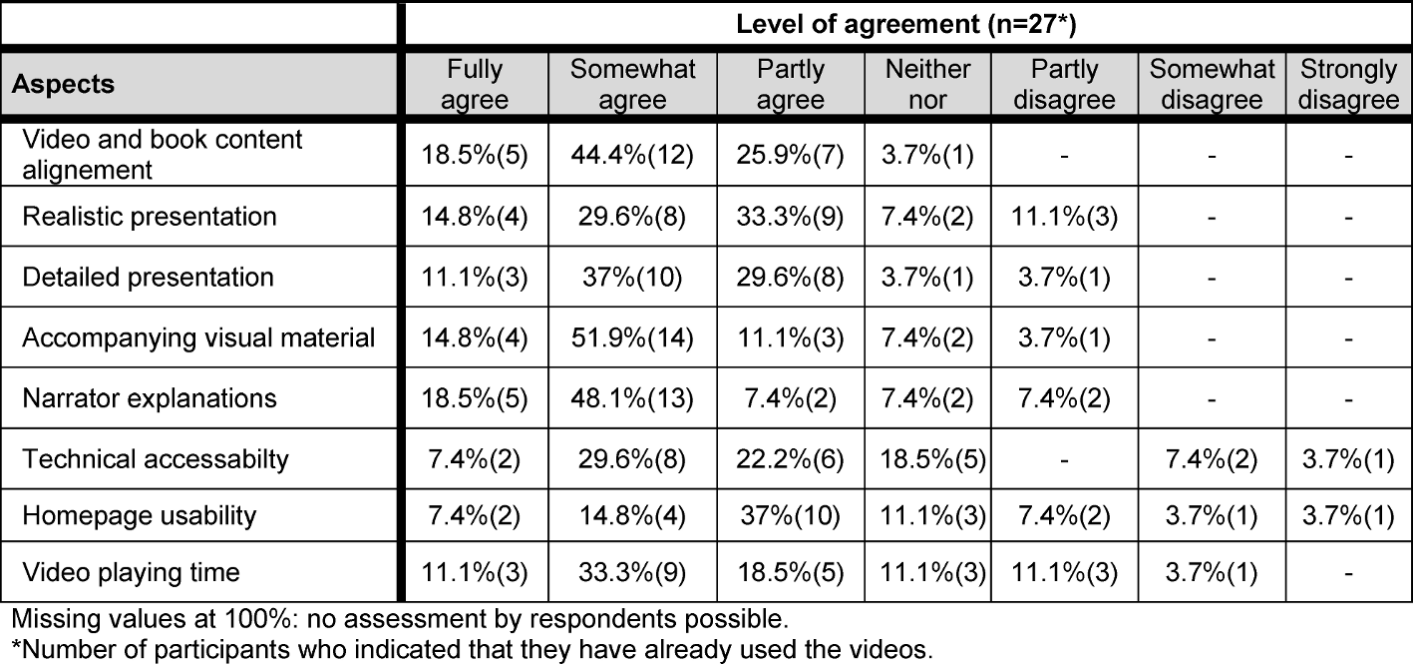
Helpful aspects of the accompanying videos as assessed by FY students (n=27).

**Table 5:**
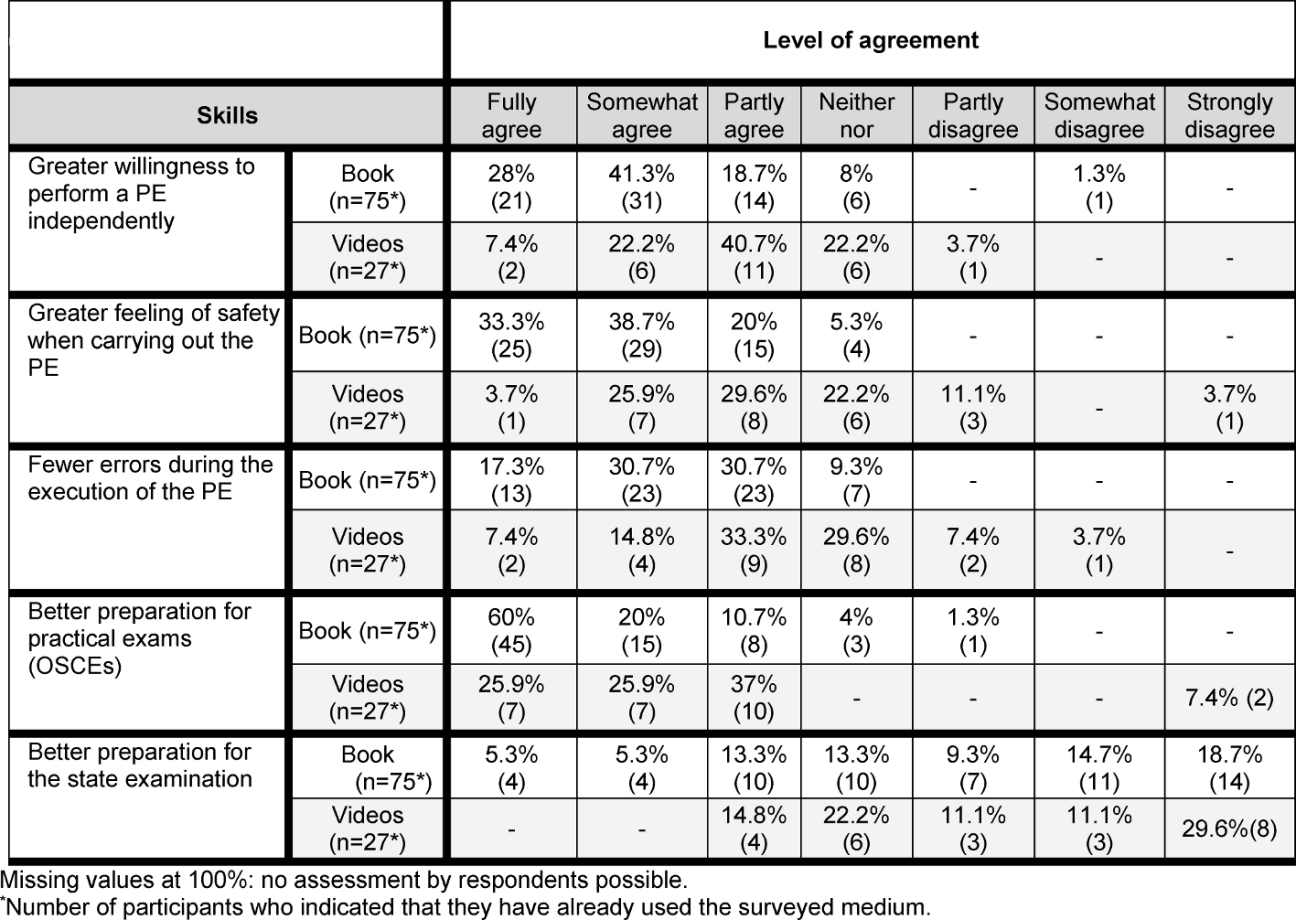
Improvement of practical clinical skills as assessed by FJ students (n=75 and n=27, respectively)

**Table 6:**
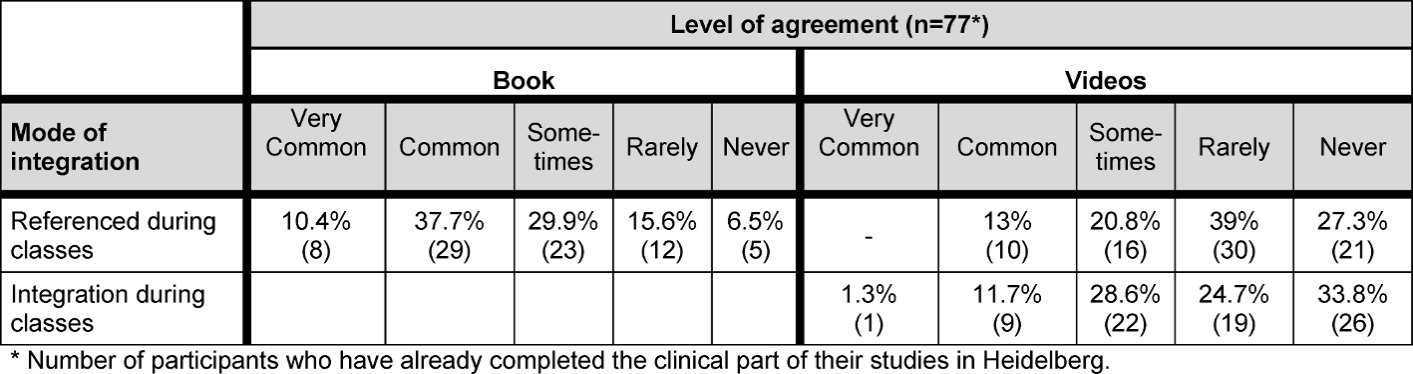
FY Students’ perceived Integration of teaching materials into the curriculum (n=77)
