# Supplementary material for: “Heidelberg Standard Examination” – Final year students' experiences with a handbook and instructional videos to improve medical competence in conducting physical examinations
Source: GMS J Med Educ. 2018 Aug 15;35(3):Doc38. doi: 10.3205/zma001184 (PMC6120156; doi:10.3205/zma001184)
Supplement: Analysis of the interviews conducted with FY students in the 2nd and 3rd tertial of FY medical education (n=10) [file JME-35-38-s-001.pdf]

Main category	Sub-catregories	N° of codes	Discription	Example quotes
**1. In what context is the manual used?**	1.1 For exam preparation	21	The book served as a reference work for practical exams in the clinical studies phase of training and for the preparation of the practical state exam.	"For the OSCEs, the [book] was absolutely perfect, I also had the feeling that if you could do what it said in there, you wouldn't have a problem in the OSCE either."<br><br>"In principle, it's a good way to prepare for many practical exams."<br><br>"You do lose the very structured procedure a bit during work on the ward, and of course it is[important] to look at it again, especially before exams, in any case before the practical exam.“
	1.2 For clinical work in the FY	20	The book is used for independent preparation and follow-up of clinical work on the ward.	"In the FY, I was in orthopedics, I used it for the examinations there that I had previously practiced during my studies, but only with checklists that were focused on the exam content. I actually only started using the book after asking myself the question: How do I actually do the [physical] examination myself, independent of exams? I used the book. “<br><br>"For actual examinations, I also looked the chapters up again beforehand to see how exactly it should be done, e.g. the abdominal examination."<br><br>"...when I then notice something in the course of the day, that I then look at it again in the evening, let it pass before my inner eye, how exactly the procedure is performed."
	1.3 For clinical studies, e.g. in classes	14	The students use the book as a guideline for the initial training of the physical examinations during student classes and to revise content.	"We always have this one or two hour ward class and always have to do a physical examination there. And we’d always have it with us because we weren't so experienced at the time. And then we'd really shimmy along there or read and look in there the day before, how to do it and so on."<br><br>"Well, that's where everyone I know, whenever we met, had the handbook with them and always did it the way it was described there."<br><br>"... and then you'd revise or take another look at it just before you went to the ward classes, what to keep in mind or something like that."
	1.4 In the context of clinical traineeships	3	It was only partially reported that the book was used in the context of the first clinical-practical traineeships	"I did a clinical traineeship in a pediatric practice and I think I took a look at the examination book there, too."<br><br>"I've done more orthopedic clinic work up to now, but I’d look in it there, too...[ ]."<br><br>"And a little for neurology, too. I actually used it in my clinical traineeship."
**2 What is the handbook used for?**	2.1 As a guideline	14	Due to the step by step explanations of the examination procedures, many reported to use the book as a helpful guide in the preparation of examinations.	"I usually find it a nice summary and you can see what’s important at a glance and what you need and in which order you should proceed.”<br><br>"...it’s a guideline along which you can shimmy."<br><br>"...for the orthopedic examinations, it starts with the shoulders and then goes to the arms and the legs. So you really can leaf through it the way you would perform an actual examination."

	2.2 To look things up	11	The book is used as a comprehensive reference work that is perceived as helpful especially in the FY and for subsequent clinical work.	"I think it's very good for clinical work. Also as a reference book, you then don't need such a huge book, but have really a compact one, which is well structured and where you can also just look something up."<br><br>"Well, I think it was during the FY that it turned out to be a wonderful reference book for me personally."<br><br>"But the book is also meant to be something to look things up later, when you are working in the clinic."<br><br>"But I really see it more as a valuable source of support, especially in the first year when you're on the wards; one then really looks things up again and reads up on stuff. So in that respect, it's really great that this has been done and has really become indispensable for assistant [doctors]."
	2.3. As a reference work	8	The book provides a standard that is described by some as an orientation aid in clinical practice, as well as providing them with a feeling of security during physical examinations.	"It's nice to hear from the assistant [doctors] what every-day clinical life looks like and it's nice to see it again in the book: in an ideal world, what would the steps look like?"<br><br>"...it's a bit like on the driving test: First you have to know what the rough rules are and then you can still see if you can change one or the other thing."<br><br>"...and then, in case of doubt - the examiners are always very different - one would also have a means where one could say: "I have done this according to this style".
	2.4 Not a pocket book	6	The book was considered as too bulky to carry around as a pocketbook.	"...just the size. You can't really take it with you in your scrub pocket. But it may not be meant to be."<br><br>"But to you carry it around in your scrubs during FY, it's a bit too thick for that after all."<br><br>"You could think about making a book with several subparts, so you could use it even more as a scrub pocket book. Because now the thing is, because you already have so much in your pockets, you still don't put it in your gown, that's why it's more of a ward desk book."
**3. Which features of the manual are described as helpful?**	3.1 The compact format and the systematic presentation of content	16	The compact and clear content presentation format was perceived as very helpful.	"It's short and concise, simply listing the essentials in bullet points. I just think it's better, I've got through it faster than this "dual series", for example. I had previously borrowed other handbooks and actually found it quite useful because it simply contains everything, in a nutshell, what you need".<br><br>"It's nice and short, you don't have to read many pages, you have everything together in one place."<br><br>"Even when you open a page and just skim across it, you've seen the most important things."
	3.2 Completeness	10	Respondents appreciated the wide variety of content.	"Whenever I looked for any specific physicals, everything I wanted to find was in there."<br><br>"Well, it used to be like this: ah, this is so much and who needs all that? But since I'm in the FY, I've realized: you actually need it all. "<br><br>"The advantages are that actually each specialist area is covered. For an orthopedic surgeon, it contains an extensive repertoire, and for an internist, everything you need to know."<br><br>"Well, what I like is that it's a lot more than I need."

	3.3 The comparison of normal findings and pathological findings	9	The interviewees often highlighted the benefit of the comparison between normal and pathological findings.	"I've always found this physiological comparison the best thing, to know what could be wrong. If you checking or examining someone, you know what could be wrong."<br><br>"...you always have such an overview: physiological and pathological findings. So, I really enjoyed working with it."<br><br>"I find it very practical to know how it should sound or feel physiologically and next to it again the column with what might sound different and what it could be."<br><br>"And what I also liked was that there were always some pathological results and what they could point to."
	3.4 The extensive illustrations	9	The detailed illustrations helped the respondents to gain a better understanding of the examination procedures.	"The pictures are also a good illustration of how the examination is carried out."<br><br>"And there are always a lot of diagrams and pictures, which I think is especially good for such orthopedic things, if you just see how high, for example, the angle that you have to measure with any mobility and such a thing."<br><br>"Sometimes it's hard to put something like that into words, so it's a good thing there are pictures in it."
**4 Which points of criticism are mentioned?**	4.1 Too extensive to be used as practical guide	15	Some interviewees complained that the examinations are too extensive and, thus, often not directly applicable to ward every-day practice.	"The initial examination here on the ward is ultimately something different from what it says in the book. Usually you don't have time to do it the way you do it."<br><br>"It is sometimes very detailed and in clinical practice it is something else."<br><br>"In this book, the PE is described in its most detailed detail - this is not always possible in everyday hospital life, we don't have the time to do it that way".<br><br>"I kind of always had it with me, but I didn't really use it. Because it is just often the case that [if] you are somewhere in the emergency room or when you are on the ward, that the examinations you have to do there are simply not as detailed as they are in the book".<br><br>"For example, the orthopedic part is extremely extensive and if you're not firm in this field, you can examine your patient’s hip for 2 hours because there are 2000 different examination possibilities."
	4.2 Lack of structure or clarity	4	The sheer volume of information was perceived as confusing and overtaxing.	"I found it a bit confusing sometimes when I was really looking for a special examination. You can look further back, on which page, but if I somehow wanted to read a very special examination again directly, then I didn't always find it immediately."<br><br>"Pediatrics really struck me down, I have to say. You can see here, it's almost 70 pages. And I also did my clinical training in the pediatrics and did the admissions and then you just go and examine the child, you know. And then 70 pages are a bit much."
	4.3 Missing properties	4	An overview of key, every- day examinations was perceived as helpful.	"...what I now often had during FY in surgery, for example, were rough neurological examinations. And in the book this is depicted on several pages, everything that there is. So maybe also a chapter: What do I do as a non-specialist in a rough neurological examination? One, two, three, four. So that it's just written on one or half a page. And I can have a look at the detailed part of the examinations

				themselves. But in that way I'm lost somehow. So, I do the neurological examinations a little bit by rule of thumb and don't know what is important. “<br><br>... "what may be missing is an assessment of how meaningful a test can be."
**5. How is the integration into the teaching perceived?**	5.1 No experienced integration of the book into classes	9	Many respondents described that they had not perceived any active integration of the book into classes and that they had not been encouraged to use it.	"Didn't I see people pointing out that we should use it."<br><br>"It wasn't always pointed out that you should use it now or that it was really integrated as a textbook. You know it exists, even if the lecturers don’t say that you should have a look in there."<br><br>"Well, from the faculty's position, I don't really remember them using it that way."
	5.2 Experienced integration of the book into classes	5	Few respondents remembered having received information about the book from lecturers. However, it was seen as a reference for the practical exams.	"...it says before the OSCEs or so: Look at the book and this is how you have to do it and this is how it is done well. This has always been integrated."<br><br>"...for the OSCEs, for the practical exams, the lecturers have already said that we should have a look at the examination book. But it's rare, it's rare."<br><br>"Well, it's almost like it serves as the expectation criteria for the OSCEs. That's perfectly matched."
**6. What is the experience with the accompanying videos?**	6.1. The videos were perceived as helpful	8	Only a few respondents had already watched the videos; they saw the films as a helpful supplement in gaining a better understanding of the handbook’s content.	"During the physical examination I took a look at it, even then in the tapping class, especially with orthopedics, the examination methods and tests are partly already complex, and therefore it is good - it is sometimes difficult when one reads such a text, one cannot imagine it correctly, such a video is certainly a good addition.”<br><br>"Well, for pediatrics, I've been watching videos, [...]. And I found it very helpful that you simply see: How did they keep it, how did they do it. I really liked that."
	6.2 No experience of use has been made so far	10	Respondents indicated that they were using films from other providers, e.g. via AMBOSS or Youtube.	"At AMBOSS, I also looked at some of them from time to time at the beginning of the exam preparation[...], they are also quite well linked. When you learn something on the site, the videos that are available are always at the bottom. So that was always quite clear and well structured, [...]."<br><br>"I used to watch all the videos on AMBOSS [for example] or something. I mean, that's the learning platform most students use. And they also have good videos of the physical exam."<br><br>"Somehow, YouTube has become the place to look."
		4	There is a general awareness that there are the accompanying videos, but the need for them did not seem to be big enough	"And every time you see that, you think, "Ah, yeah, you should take a look at that.” But somehow it just never happened."<br><br>"I think I had that in mind too, but I've never looked at it."<br><br>"I know the videos exist and I've actually been watching them for ages, but I just can't get into it because it’s just slipped right to the back of my head. “

		3	Web-access was described as too complicated	"Back then, it was even harder to log in, so that's why I didn't think it was really very convenient."<br><br>"I must confess, I would have to check first, where the videos are deposited, so I just know about the pediatric videos in Moodle, but if there is now an extra page with videos for standard examinations or so, where really all the videos can be found - Well, I honestly do not know that. So, I would not look for it now. Unless such a page would be better known and well-structured. "<br><br>"The moment you have to log in again! You just don't do that."
